# Indigenous knowledge and use of medicinal plants for ethnoveterinary within the North West Province, South Africa

**DOI:** 10.3389/fvets.2023.1273562

**Published:** 2023-11-22

**Authors:** Rendani Victress Ndou, Simeon Albert Materechera, Mulunda Mwanza, Wilfred Otang-Mbeng, Mooki Fabridge Ijane

**Affiliations:** ^1^Centre of Animal Health Studies, North West University, Mmabatho, South Africa; ^2^Indigenous Knowledge Systems Centre, North West University, Mmabatho, South Africa; ^3^Faculty of Natural Sciences and Agriculture, School of Biology and Environmental Sciences, University of Mpumalanga, Mbombela, South Africa

**Keywords:** African traditional medicine, ethnoveterinary medicine, animal health, indigenous knowledge, medicinal plants

## Abstract

Ethnoveterinary medicine (EVM) has been used by local South African communities for centuries. However, the knowledge of EVM is under threat due to the influence of Western medicine and acculturation. This study aimed to document the knowledge of ethnoveterinary medicinal plants in four villages within the Mahikeng Local Municipality to help preserve this valuable knowledge. The study used a qualitative research approach and targeted practitioners and holders of ethnoveterinary knowledge in four villages. Participants were recruited using key informants and snowball techniques, and in-depth interviews were conducted using semi-structured interview schedules. The data collected was analyzed thematically, and herbarium specimens were prepared from medicinal plants during field walks and sent to the South African National Biodiversity Institute (SANBI) for identification. The study documented the use of thirty-one medicinal plant species, with *Senna italica* (sebetebete) emerging as the most frequently used species for multiple animal health indications. The most common diseases treated using EVM were gala (general malaise due to bile reflux) and retained placenta. Participants strongly agreed on the cultural significance of plants for treating five animal diseases, including coughs and diarrhea. Although the study revealed a high level of trust in ethnoveterinary medicine among participants, they expressed concern regarding the loss of this knowledge due to the influence of Western medicine and acculturation. The study concluded that the indigenous knowledge and use of ethnoveterinary medicine was prevalent among livestock owners in the communities of the North West Province, and more studies need to be conducted in other areas to preserve this valuable knowledge.

## 1 Introduction

Ethnoveterinary medicine (EVM) is a term used to describe the traditional knowledge and practices of various communities in South Africa and other parts of the world, caring for the health and productivity of their animals (1). In Africa, the knowledge is a part of African traditional medicine (ATM) (2). It involves a wide range of practices such as the use of plants, minerals, and indigenous remedies to treat and prevent animal diseases (3, 4). Animal health problems are diverse and can lead to significant production losses for farmers due to morbidity and mortalities (5). Before the emergence of Western medicine, EVM served as the primary animal health solution for most African farmers (6). At present, EVM is still an important aspect of rural South African communities and plays a vital role in animal healthcare, particularly in areas where modern veterinary services are limited (7).

During the colonial and apartheid periods in South Africa, the authorities attempted to eliminate African traditional medicine, including EVM, by labeling it as witchcraft and magic and imposing bans on it among indigenous communities (8). However, despite these efforts, EVM has survived and persisted over time in many regions of South Africa (9) as a crucial component of primary animal healthcare management (7, 10). In some instances, some farmers prefer EVM over Western veterinary medicine due to their belief that it is more effective (11). In addition, EVM is more readily available and cost-effective than Western medicine (12), making it a preferred choice for many farmers. These factors, coupled with the Western world’s search for sustainable alternatives to Western medicine, have led to a renewed interest in ethnoveterinary medicine and the recognition of its value (7).

Ethnoveterinary medicine is still widely used by farmers in many parts of South Africa as a crucial component of primary animal healthcare management (7, 10). Some farmers prefer EVM over Western veterinary medicine due to their belief that it is more effective (11). In addition, EVM is more readily available and cost-effective than Western medicine (12), making it a preferred choice for many farmers. These factors, coupled with the Western world’s search for sustainable alternatives to Western medicine, have led to a renewed interest in ethnoveterinary medicine and the recognition of its value (7).

The renewed interest in EVM, has led to various exploratory surveys conducted globally to document the knowledge of EVM to preserve such knowledge (13). The South African government, through the National Indigenous Knowledge Systems Office of the Department of Science and Technology, supports the preservation and promotion of indigenous knowledge, including EVM (14). However, there are very few surveys in South Africa that document the knowledge of EVM, with most of these surveys conducted in the Eastern Cape and Limpopo Province (7, 13, 15). Though, livestock production is a significant source of income for rural communities in the North West Province, particularly in the Mahikeng Local Municipality, there’s no record of any studies conducted on the use of ethnoveterinary medicinal plants by local practitioners. This study aimed to document such practices in villages within the Mahikeng Local Municipality, North West Province with the objective to preserve this valuable knowledge.

## 2 Materials and methods

### 2.1 Study area

**Figure 1:**
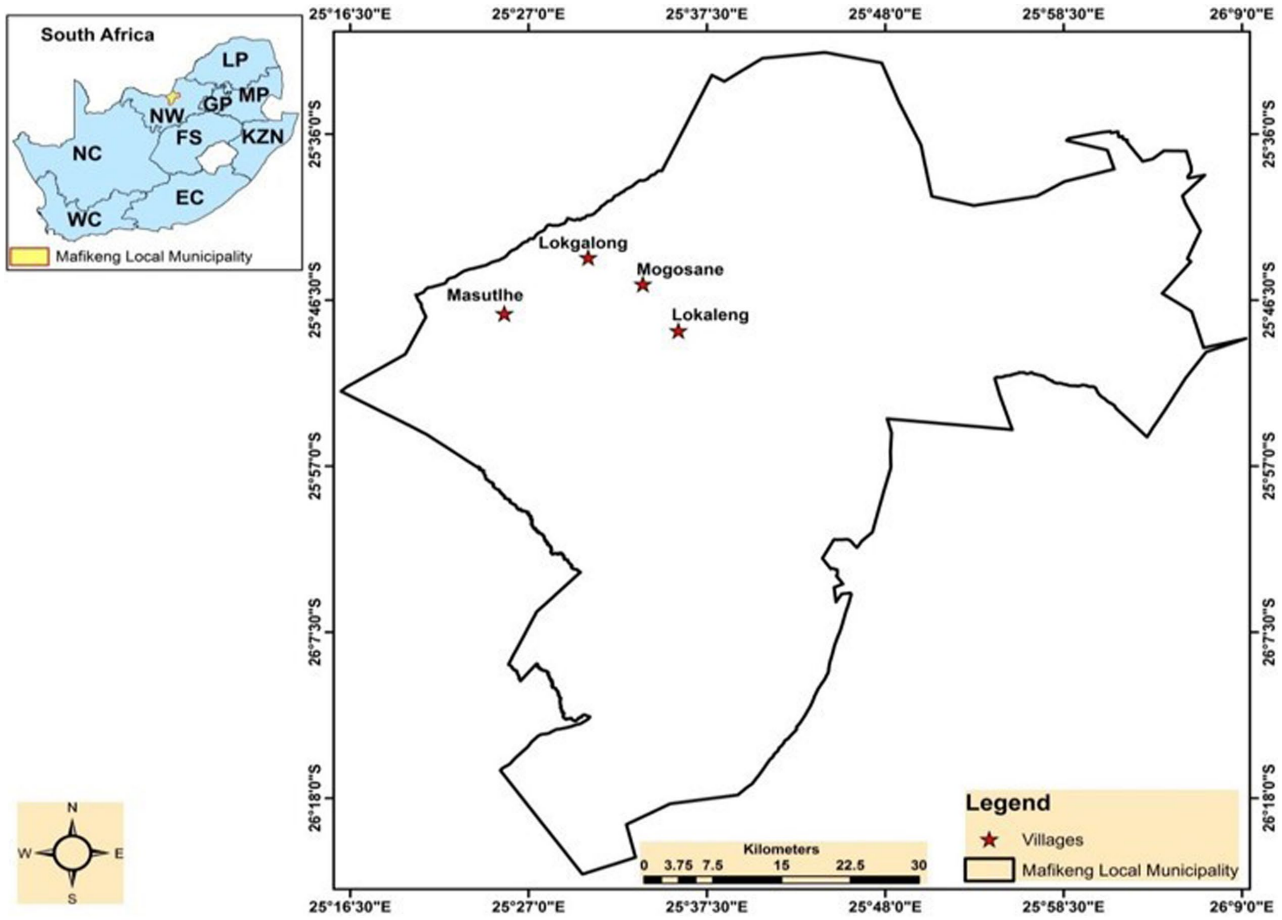
The location of Lokaleng, Mogosane, Lokgalong and Masutlhe villages within the Mahikeng Local Municipality and location of the North West (NW) Province within South Africa.

The study was conducted in four villages located within the Mahikeng Local Municipality of Ngaka Modiri Molema District Municipality in the North West Province *viz* Lokaleng, Mogosane, Lokgalong, and Masutlhe (Figure 1). The villages included in this study are located on traditional lands governed by traditional leaders (known as *Dikgosi*). Nearly 100% of the population in each village is Black African, with the majority being Setswana speakers at an average of 90.3%. Masutlhe village covers an area of 80.93 km^2^, with a population of 3,085 and 811 households. Lokaleng village has a total population of 2,661 and covers an area of 80.93 km^2^, with 715 households. The population of Lokgalong village is the smallest among the four villages, with only 233 people (7.13 per km^2^) and an area of 32.67 km^2^. Lastly, the village of Mogosane has an area of 34.41 km^2^, a population of 1,927, and 433 households (16). The villages are situated North West of the town of Mahikeng and are under the traditional leadership of the Barolong Boo-Ratshidi, an ethnic group of the Batswana which is found in both South Africa and Botswana (17). Mahikeng town is the headquarter for the Barolong Boo Ratshidi people and also the Capital of the North West Province. Barolong boo Ratshidi, like any other Batswana ethnic group, has a strong farming history and presence. In rural areas of the Mahikeng Local Municipality, agriculture is the main economic activity and the main livestock commodities are cattle, sheep, goats and chicken (18). They depend on livestock for daily needs such as milk, meat, and draught power and important cultural ceremonies such as weddings and funerals. Livestock symbolizes wealth, strength, and stability to Barolong (17). The climatic condition in Mahikeng Local Municipality is typical semi-arid savannah with a mean annual summer rainfall of 500 mm with temperatures that range from 17° to 31°C (62° to 88°F) in the summer and from 3° to 21°C (37° to 70°F) in the winter (19).

### 2.2 Unit of analysis and target population

An individual EVM practitioner and knowledge holder were the unit of analysis. The target population consisted of members of the communities who had some knowledge of ethnoveterinary medicine and, thus, referred to as ethnoveterinary practitioners (EVPs). The EVPs included livestock farmers and traditional healers known and recognized by communities in each of the four study villages.

### 2.3 Sample size and sampling procedure

The sample consisted of twenty-one ethnoveterinary practitioners (EVP) from the four villages. The identification and recruitment EVPs were done through snowball sampling (20). In brief, after each interview, participants were requested to refer the next participant for the study, and the person was then approached and recruited to participate. The names of the first participants in Lokaleng (*n* = 6), Lokgalong (*n* = 7), and Mogosane (*n* = 4) villages were provided by the translators, while in Masutlhe village (*n* = 4), the name of the first participant was provided by the office of the *Kgosi’*s (Chief). The snowballing continued until no new ethnoveterinary practitioner could be identified (20).

### 2.4 Data collection techniques

The data collection tool consisted of a semi-structured interview schedule with closed and open-ended questions. Data was collected using semi-structured face-to-face, in-depth interviews with participants. The interviews were conducted in the local Setswana language with the assistance of translators. Information provided by participants during the interviews was captured on a voice recorder. Although the researchers originally intended to conduct individual interviews with each participant, they ended up conducting most of the interviews in groups of two or more people, following the African community style. To ensure participants’ comfort, all interviews were conducted at a place of their choice, including their homes and informal gathering places. To authenticate the data, member checking was done with the participants after the data was transcribed to verify accuracy of the information (21, 22).

### 2.5 Collection and identification of herbarium specimens

Medicinal plants mentioned during interviews were identified by participants during field walks in grazing, crop, and open lands in the different villages and the open fields at the North-West University. The field walks aimed to observe live plants *in situ* and collect plant samples for botanical identification. The whole plant or plant parts were collected and pressed to prepare the herbarium specimens. Thirty herbarium specimens were botanically identified at the South African National Biodiversity Institute (SANBI) and one herbarium specimen was botanically identified at the AP Goossens Herbarium, Potchefstroom campus, North-West University.

### 2.6 Data analysis

#### 2.6.1 Qualitative data analysis

Data collected during interviews were first transcribed from the voice recorders and translated from Setswana into English with the assistance of the translators and then transferred to a Word document. Thematic analysis was manually done on the data according to Braun and Clarke (23).

#### 2.6.2 Calculation of informant consensus factor (F_IC_) for data on medicinal plants

The informant consensus factor (FIC) was calculated for some diseases to determine culturally significant medicinal plants in the study area (24–26). The diseases that were selected were those that were frequently mentioned by participants and had multiple treatment options using medicinal plants. The informant consensus factor (F_IC_) was calculated as follows:

(1) $$F_{IC}=Nur-Nt/\left(Nur-1\right)$$

where, Nur = number of used reports from informant for a particular plant-use category; Nt = number of taxa or species used for that plant use category for all informants; F_IC_ Values ranged between 0 and 1, where ‘1’ indicates the highest level of informant consent (24).

### 2.7 Ethical considerations

Ethical approval for the study was obtained from the North West University Research Ethics Committee (Ethics number: NWU-00554-16-A9). Additionally, the researcher followed traditional protocols and sought permission from the traditional leadership (*Dikgosi*) in the four villages before conducting research among the people. All village Chiefs allowed the researcher to conduct the study in their respective villages. Prior informed consent was obtained from all participants before the interview. The participants were informed about the study and how the data collected would be handled. The consent form also clarified that the data collection process was voluntary, and that personal information would not be published without their permission. During the interviews, the researchers followed Batswana cultural protocols, including appropriate dress codes, time observation, and respect for participants. This required the researchers to immerse themselves in the culture of the communities to gain an understanding and appreciation of the cultural values, learn the cultural “dos and don’ts” of the Batswana, and improve their knowledge of the Setswana language.

## 3 Results and discussion

### 3.1 Demographic and other characteristics of participants in the study

All participants in the study were farmers, and only one was also a traditional healer. Most participants were men (85.7%) above the age of 50 years (Table 1). When asked about their farming experience, all participants indicated they were “born into farming.” All the participants were long-time residents of their respective villages, and all were Batswana.

**Table 1:** Demographic and other characteristics of the participants.

Participant	Age range	Gender	Farming enterprises	Source of EVM knowledge
1	60–70	Female	O, C, B, FRC	Ancestors and parents
2	60–70	Male	O and C	Parents
3	20–30	Male	C	Grandfather
4,5,6,7	30–70	Males	B, G, FRC	Parents and relatives
8	40–50	Male	O, C, FRC	Parents and relatives
9	70–80	Female	B, C, O, FRC	Father
10, 11,12	60–100	Males	B, C, O, FRC	Parents and grandparents
13,14, 15	40–70	Female (13) and two males	B, C, O, FRC	Parents and relatives
16, 17	60–70	Male and female	B, C, O, FRC	Parents and grandparents
18, 19	60–70	Males	B, C, O, FRC	Parents and grandparents
20, 21	70–90	Male and female	B, C, O, FRC	Parents and grandparents

O, Ovine; B, Bovine; C, Caprine; FRC, free range chicken.

### 3.2 Knowledge of plants used for ethnoveterinary medicine

A total of 31 medicinal plants were documented for 25 animal health indications (uses), including fracture repair, protection of livestock after the death of a family member (sefifi), uterine cleansing, retained placenta, gala (general malaise due to bile reflux and gallsickness in cattle), diarrhea of ruminants, gastrointestinal parasites, blood cleansing, pain, maintenance of pregnancy after a previous abortion in cows, and reproductive efficiency/fertility enhancement as shown in Table 2. Many of the plants documented have been reported in other studies in South Africa. However, most of the treatment indications are different, which represents a new potential for the use of the plants. Additionally, a novel finding was that 10/31 (32.3%) medicinal plants were reported for the first time for ethnoveterinary medicine in South Africa. The plants were *Amaranthus lividus, Dicoma galpinii, Kleinia longiflora, Cadaba aphylla, Cucumis myriocarpus,* a *Thesium* spp.*, Acrotome inflata, Asparagus nodulosus, Cannabis sativa,* and *Euphorbia serpens*.

**Table 2:** Plant species and families used in ethnoveterinary medicines are arranged, according to their local/Setswana names, medical indications, frequency of mention (F) and methods of preparation, and route of administration.

Family, scientific name, and voucher number	Setswana name	Medical indication(s)	F^b^	Method of preparation and route of administration
**Amaranthaceae** *Amaranthus lividus,* NRV^a^ 25.	Modinakana	Blood cleansing in cattle. Wounds not healing in livestock.	4 4	Oral, leaf infusion.
**Amaryllidaceae** *Boophone disticha* (L.f) Herb, NRV^a^ 22.	Leswama	Fracture in cattle. Post-abortion in cattle. Retained placenta in cattle. Maintenance of subsequent pregnancy after abortion in cattle.	1 1 5 1	Fractures, bulb scales are wrapped around the fracture line before applying the splint made of *Acacia Karoo* bark. For all other diseases and conditions, oral, bulb decoction.
**Apocynaceae** *Gomphacarpus fruticosus* (L.) Aiton f. *subsp. Fruticosus,* NRV^a^ 36.	Sebogamashi	Retained placenta in cattle. *Gala*^c^ in goats and chickens. Respiratory diseases in chicken.	1 2 2	Oral, root decoction for goats and cattle. For chickens, the root decoction is poured into the drinking water.
**Asparagaceae** *Asparagus nodulosus* (Oberm) J.P.Lebrun 7 Stock, NRV^a^ 11.	Radipolwane/ polopolwane	Eye infection in livestock. Retained placenta in cattle.	2 1	Eye infection, warmed root juice eye application. Retained placenta, oral, root decoction.
**Asphodelaceae** *Aloe zebrina* Baker, NRV^a^ 07.	Kgophane	Ripening of abscess in livestock. Fleas in poultry. Gastrointestinal parasites in poultry. General health in poultry. Gala in chicken.	2 1 1 3 3	Abscess, heated topical leaf application: The leaves are put on fire to heat them up and then placed on a hard abscess to “ripen,” and after it becomes soft, it will be lanced and cleaned. Chicken diseases: Leaves are put in drinking water.
**Asphodelaceae** *Bulbine abyssinica* NRV^a^ 30.	Makgabenyana	Blood cleansing in livestock. Internal sores in livestock.	4 4	Oral, root infusion: Roots combined with roots of *S.lichtensteinii* and *W.somnifera.*
**Asteraceae** *Artemisia afra* Jacq. ex Willd. var. *afra,* NRV^a^ 34.	Lengana	Cough in cattle.	4	Oral leaf infusion: Leaves combined with roots of *H.caespititium*.
**Asteraceae** *Dicoma galpinii* F.C. Wilson, NRV^a^ 18.	Tlhlonya	Diarrhoea in cattle and goats. *Gala*^c^ in all animals. Blood cleansing in cattle. Pain in cattle. After abortion. Cleansing the uterus after dystocia. Stomach pain in livestock.	4 4 2 2 3 3 2	Diarrhoea and *gala*, oral, root infusion, mixed with root of *S.italica.* Blood cleaning and pain, oral, root decoction, combined with roots of *Z.zeyheriana, S.italica* and *C.aphylla*. Abortion and dystocia, oral, root infusion. Stomach pain, oral root decoction.
**Asteraceae** *Helichrysum paronychioides* DC, NRV^a^ 15.	Phateyangaka	Coughs in cattle. Blood cleansing in cattle. Pain in livestock. Diarrheoa in calves.	7 2 2 2	Coughs, oral, root infusion, with leaves of *A.afra.* Blood cleansing and pain, oral, root decoction, combined with roots of *S.panduriforme* and *W. somnifera*. Diarrhoea, oral, root decoction combined with roots of *Z.zeyheriana*.
**Asteraceae** *Kleinia longiflora* DC, NRV^a^ 35.	Mosiama	Fracture in livestock. Protect livestock after death in the family (sefifi).	1 3	Metaphysical fracture repair (*Go tlhabela thobega*). Sefifi, the plant is put in water and spread in the kraal.
**Asteraceae** *Tarchonanthus camphoratus* L, NRV^a^ 16.	Mohatlha	To prevent cold in small stock.	2	Leaves put in drinking water.
**Brassicaceae** *Cadaba aphylla* (Thunb.) Wild NRV^a^ 23.	Monnamontsho	Blood cleansing in cattle. Pain in cattle.	2 2	Oral, root decoction: Combined with roots of *Z.zeyheriana, S. italica* and *D.galpinii*.
**Cannabaceae** *Cannabis sativa L.* NRV^a^ 38.	Motekwane	Anthelmintic in donkeys and horses.	1	Oral, leaves decoction.
**Cucurbitaceae** *Cuminis myriocarpus* Naudin *subsp myriocarpus,* NRV^a^ 05.	Monyaku	To induce abortion in dogs. To induce vomiting in dog when there was general malaise (gala).	1 2	Oral, fruit juice infusion with milk. Seeds are highly toxic and should not be administered.
**Euphorbiaceae** *Croton gratissimus* Burch. *var. gratissimus,* NRV^a^ 33.	Moologa	Fertility enhancement.	4	Leaves are dried, crushed and mixed with supplement feed or spread in the kraal.
**Euphorbiaceae** *Euphorbia serpen* Kunths, NRV^a^ 27.	Luetsane	Blood cleansing in cattle.	2	Oral, root decoction, roots mixed with a bit of bulb of *D sanguinea*.
**Fabaceae** *Elephantorrhiza elephantina* (Burch) Skeels., NRV^a^ 20.	Bosetsana	Blood cleansing in cattle.	1	Oral, rhizome infusion, oral route: The rhizome is crushed and put into water.
**Fabaceae** *Indigofera cryptantha* Benth. ex Harv. var. cryptantha, NRV^a^ 10.	‘kofi”	Calf diarrhoea.	1	Oral, root decoction.
**Fabaceae** *Senna italica* Mill. subsp. *arachoides* (Burch) Skeels, NRV^a^ 06.	Sebetebete	*Gala*^c^. Diarrhoea in cattle and sheep. Retained placenta in cattle. After abortion in livestock. Gastrointestinal parasites in ruminants. General health in livestock. Blood cleansing in cattle. Pain in livestock.	19 20 3 1 1 1 2 2	General preparation, whole plant or root decoction, given orally. Can also be combined with *Dicoma galpinii* in a root infusion. Blood cleansing and pain, oral root decoction: Combined with roots of *Ziziphus zeyheriana, Cadaba aphylla* and *Dicoma galpinii*.
**Fabaceae** *Vachellia karroo* (Hayne) Banfi & Gallaso, NRV^a^ 09.	Mookana	Fracture repair in livestock (Go tlhabela thobega). Fracture repair.	3 3	G*o tlhabela thobega,* the root powder is included in a metaphysical fracture repair procedure. The bark is used as splints to stabilise fracture line.
**Hyacinthaceae** *Drimia sanguinea* (Schinz) Jessop NRV^a^ 12.	Sekaname	Snake bite in cattle. Build immunity against heartwater in cattle and small-stock. To build immunity against *Drimia sanguinea* for livestock. Blood cleansing in cattle.	4 2 1 1	Snake bite, oral, bulb infusion (a small portion). Heartwater, the bulb is dug out and put into the kraal and animals trample over it so that they get immunity against the plant (exposure treatment). Immunity against *D.sanguinea* the bulb is crushed and mixed with water and then sprinkled in kraals. Alternatively, the bulb is placed in the kraal for animals to trample over (exposure prophylaxis). Blood cleansing, oral, bulb decoction (a small portion) combined with roots of *E.serpens*.
**Iridaceae** *Babiana hypogaea* Burch NRV^a^ 14.	Thuge	Diarrhoea in goats.	1	Oral, crushed tuber crushed infusion.
**Lamiaceae** *Acrotome inflata* Benth NRV^a^ 19.	Mogato	Wounds and abscess in livestock.	4	Oral, root infusion.
**Lamiaceae** *Teucrium trifidum* Retz. NRV^a^ 32.	Lethe la noga	Maintenance of pregnancy after cow had aborted.	1	Oral, whole plant is crushed and placed in water.
**Rhamnaceae** *Ziziphus mucronata* Willd. subsp. *mucronata,* NRV^a^ 28.	Mokgalo	Abscess ripening in livestock.	2	Crushed leaves and soft branches poultice, placed on a hard abscess.
**Rhamnaceae** *Ziziphus zeyheriana* Sond, NRV^a^ 26.	Sekgalofatshe/Mokgalofatshe	Blood cleansing in cattle. Pain in livestock. Calf diarrhoea.	2 2 2	Blood cleansing and pain, oral root decoction, combined with roots of *C.aphylla*, *S.italica* and *D.galpinii* roots. Diarrhoea, oral, root decoction combined with root of *H caespititium* or root decoction combined with root of *G.flava*, The roots of both plants are crushed and boiled together.
**Santalaceae** *Thesium* spp., NRV^a^ 08.	Motlhogapele	Diarrhoea in calves and cows. Gastrointestinal parasites in calves.	2 1	Oral, whole plant decoction or root decoction.
**Solanaceae** *Solanum campylacanthum* Hochst. ex A.Rich. subsp.*panduriforme* (Drege ex Dunal) J, NRV^a^ 21.	Tholwane e nyane	Blood cleansing in cattle. Pain in cattle.	2 2	Oral, root decoction: combined with *H.caespititium* and *W.somnifera* (root).
**Solanaceae** *Solanum lichtensteinii Willd.,* NRV^a^ 03.	Tholwane	Blood cleansing in livestock. Internal sores caused by gastrointestinal parasites in livestock.	4 4	Oral, root infusion combined with roots of *B. abyssinica* and roots of *W.somnifera*.
**Solanaceae** *Withania somnifera (L.) Dunal,* NRV^a^ 31.	Modikasope	Internal sores in cattle. Blood cleansing in cattle. Pain in cattle.	4 2 2	Internal sores, oral tuber infusion combined with roots of *S.lichtensteinii* and *B.abyssinica*. Blood cleansing and pain, oral, tuber decoction, combined with roots of *H.caespititium* and *S.panduriforme*, oral, alternatively, tuber decoction: combined with roots of *Helichrysum caespititium* and *Solanum campylacanthum*.
**Tiliaceae** *Grewia flava* DC., NRV^a^ 17.	Moretlwa	Calf diarrhoea.	2	Oral, root decoction combined with root of *Z.zeyheriana* (described under *Z.zeyheriana*).

a NRV = Ndou RV for voucher number.

b F = frequency of mention of the plant.

c Gala = general malaise due to bile reflux in chicken, sheep and goats and can include gallsickness (Anaplasmosis) in cattle.

The medicinal plants recorded in this study belonged to seventeen plant families, and the most common plant family was Asteraceae, with six plant species, followed by Fabaceae with four plant species, and Solanaceae, with three plant species. These families are among those found to be popular in South African ethnoveterinary medicine according to reviews by McGaw and Eloff (1), McGaw et al. (7), Selogatwe et al. (13), Chakale et al. (15). The similarities in the findings those reviews, and the current study demonstrate that most South African indigenous communities have a consensus on the medicinal value of certain plant families. However, it is to be noted that in those reviews, the Fabaceae family had the highest number of plant species used in EVM, whilst in this study the family came second to Astreraceae. In three other studies published of EVM of Batswana in the North West Province by Chakale et al. (10), Van der Merwe et al. (22) and Moichwanetse et al. (27), Fabaceae family was the also among the most popular. According to McGaw et al. (7), the dominance of Fabeceae family in ethnoveterinary medicine of South Africa is attributed to the abundance of the family in South Africa (7). Furthermore, Chakale et al. (10) suggest that the popularity of certain plant families may be influenced by traditional beliefs, ease of harvesting and storage, as well as evidence of previous efficacy.

### 3.3 Utilization of medicinal plants for ethnoveterinary

**Figure 2:**
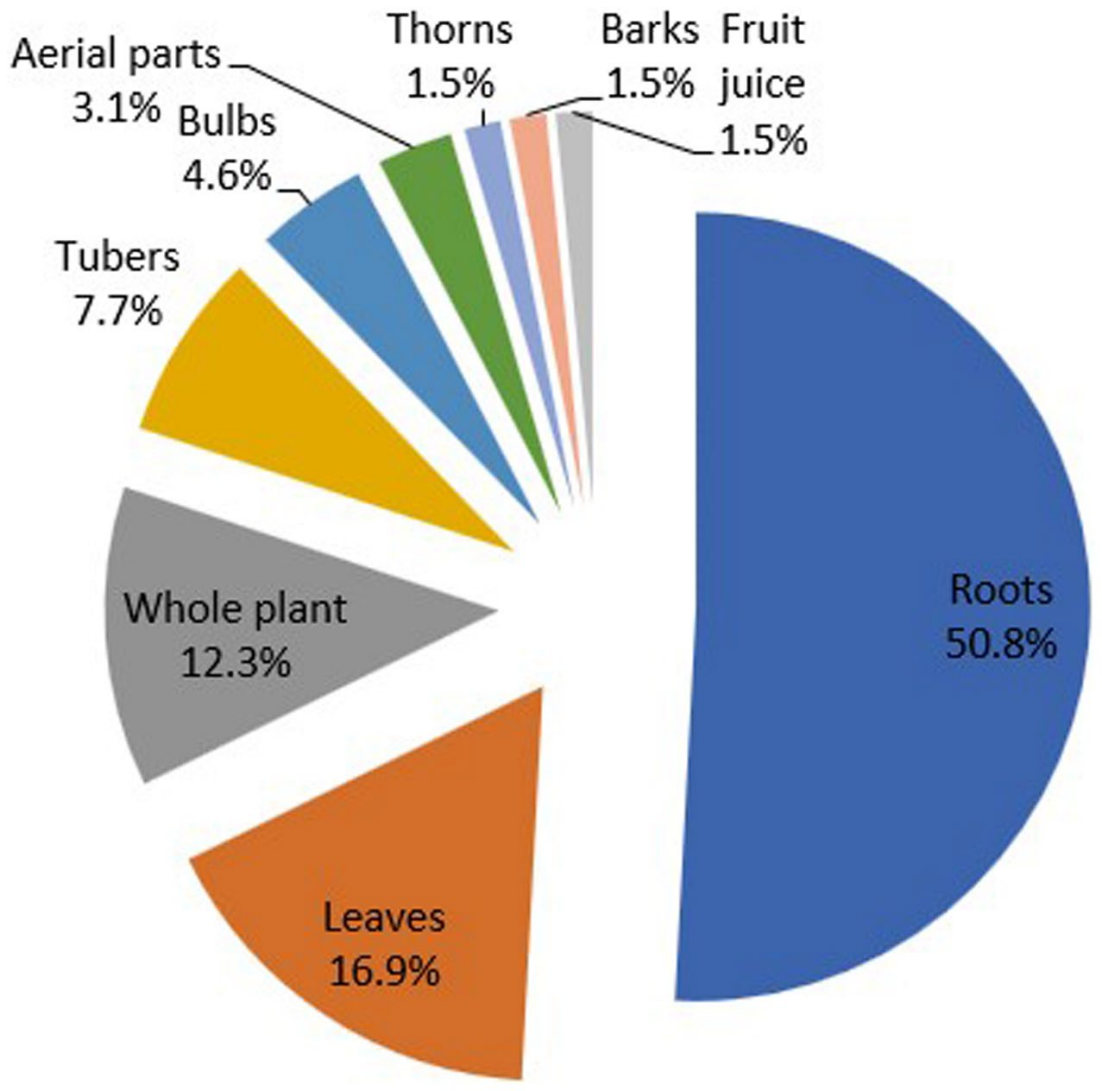
Distribution (%) of plant parts used in the preparation of remedies or other applications.

The study revealed that various plant parts were utilised in the preparation of ethnoveterinary remedies, including aerial parts, thorns, fruit juice, bark, leaves, roots, bulbs, tubers, and whole plants, as illustrated in Figure 2. Out of a total of sixty-five remedy preparation methods that were documented, roots (50.8%) are the most used plant parts, followed by leaves with 16.9%. The use of fruit juice, thorns, and barks is rare in the study area. In addition, most remedies are prepared as decoctions and administered orally (41.5%). The second most popular method of preparation and administration was oral infusions (37%). Other administration forms included topical, exposure, splinting, metaphysical application and feed additives. The preference for the use of roots to make remedies as reported here was also documented among the Batswana of Madikwe and Dinokana in studies conducted by Van der Merwe et al. (22) and Moichwanetse et al. (27), respectively. This may indicate a cultural preference for roots among Batswana people. However, another study by Chakale et al. (10) found that leaves were the most used, although the use of roots came in a close second. These findings contradict those by Masika and Afolayan (28) and Maphosa and Masika (29) who reported the high use of leaves for ethnoveterinary remedies in communities in the Eastern Cape.

### 3.4 Multiple treatment indications for medicinal plants

**Figure 3:**
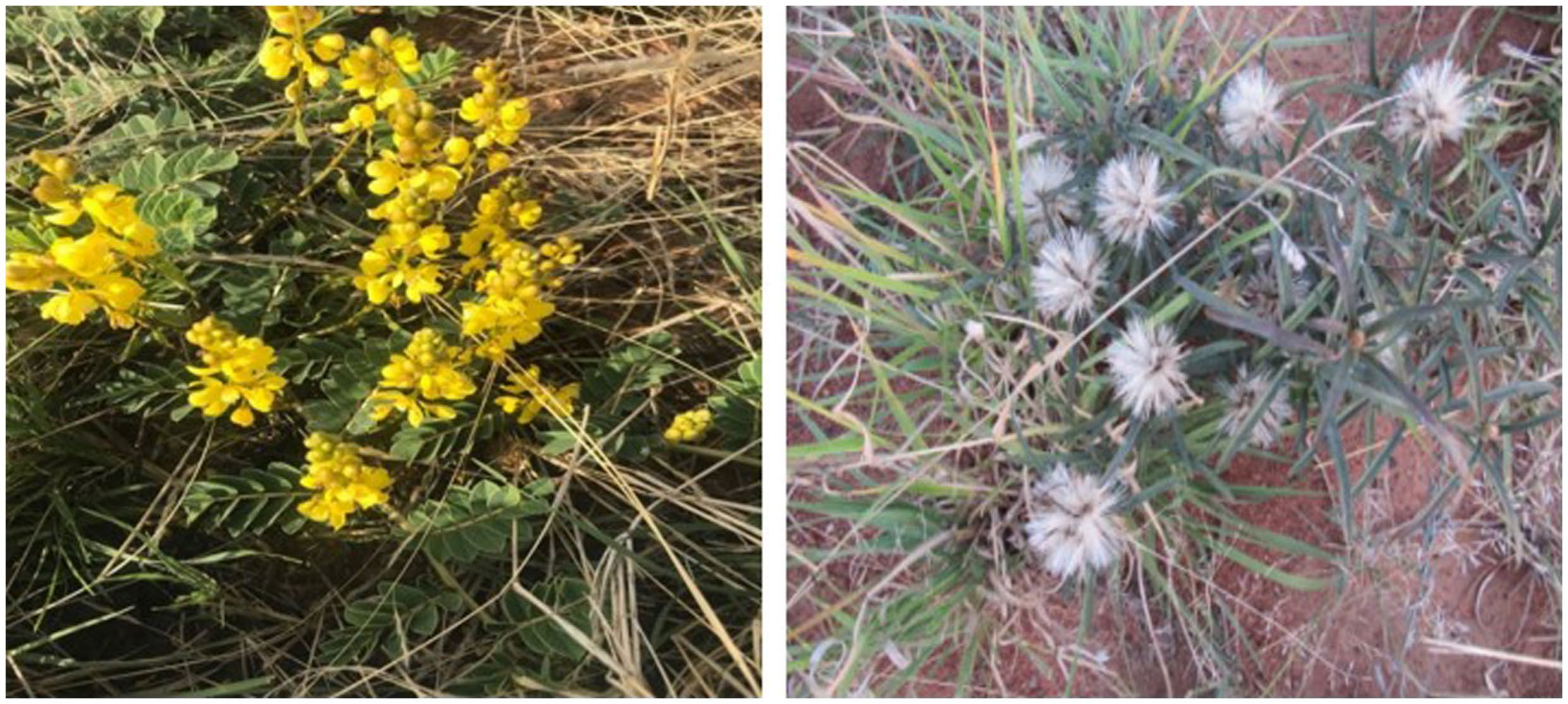
*Senna italica* with flowers on the left and *Dicoma galpinii* on the right. Source: Researcher’s data.

Fifteen out of the thirty-one medicinal plants in the study had multiple uses as single-plant or poly-plant remedies. Single-plant remedies with multiple indications included *Senna italica, Dicoma galpinii*, *Aloe zebrina*, *Drimia sanguinea, Boophone disticha,* Thesium sp., *Asparagus nodulosus,* and *Gomphacarpus fruticosus*. *Senna italica* (Figure 3) had the highest number of indications (7) in animal health care. The plant was mentioned for treating animal health problems such as *gala* (general malaise due to bile reflux), diarrheoa in calves, cows, and sheep, retained placenta, abortions, gastrointestinal parasites, general health, and blood cleansing. A participant best expressed the importance of this plant among participants in the study area in the following excerpt: *“Sebetebete ke sona molemo wa Setswana,”* expressing the importance of the plant to Batswana people. The other evidence of the importance of *sebetebete* in the study area is that it is almost always mentioned first by participants during interviews. This indicates that it is probably the first plant in participants’ minds when issues of traditional animal healing are discussed. In addition, many participants also indicated that it was also a great medicine for *gala* in humans. These narratives are best represented by the words of a participant as follows: “*Sebetebete is great for ‘gala’ in all livestock and even yourself. When you have ‘gala,’ you prepare it and drink it. You will feel the ‘gala’ going down and you will release it in a form of diarrhoea.”*

Another plant with many treatment indications (5) is *Aloe zebrina* (kgophane). The plant is used for abscess ripening in ruminants. However, it is also the most popular plant for treating poultry diseases. The indications for the plant in poultry include fleas, helminths, general health, and *gala*. *Dicoma galpinii* (Figure 3) is another plant with five ethnoveterinary indications, including diarrhoea, gallsickness, blood cleansing, uterine cleansing after abortion, and stomach pain. Other plants, including *Drimia sanguinea* (sekaname) and *Withania somnifera* (modikasope), also had a higher number of indications (4) but were also included in poly-plant mixtures with other plant species. Eight poly-plant remedies with two, three, or four plant combinations were documented to treat various diseases, and some poly-plant remedies have multiple indications. The pluralism in treatment found in this study is not unique in the South Africa context, similar findings of were reported by Van der et al. (22) where approximately twenty of the plants species out of forty-five recorded, had more than one ethnoveterinary indication. Also, Masika and Afolayan (28), found that thirteen of the thirty-six plant species recorded had two or more indications. This phenomenon has been reported in most studies conducted in South Africa that have documented ethnoveterinary medicine as reported in different reviews (1, 7, 15) and studies (22, 27). The pluralism in treatment indications means that indigenous communities have discovered the multiple medical effects of certain plants because medicinal plants possess different compounds that have different effects when applied to animals.

The poly-plant remedy is referred to as *pitsa* in the Setswana language. Plants such as *Euphorbia serpens*, *Cadaba aphylla, Solanum campylacanthum, Solanum lichtensteinii, Withania somnifera, Grewia flava* and *Bulbine abyssinica* are only used as part of poly-plant remedies. The documenting of several poly-plant remedies in this study confirmed the cultural importance of *pitsa* among Batswana people and the purpose is to increase the curative potency of remedy (10). This finding is similar the three other studies among the Batswana (10, 22, 27). However, poly-plant remedies are not very common among the Vatsonga and Vhavenda of Limpopo Province as few poly-plant remedies are documented (12, 21, 30). However, poly-plant combinations are found to be a common practice in the Eastern Cape (9, 28, 29, 31).

### 3.5 Cultural significance of medicinal plants used in the area

**Figure 4:**
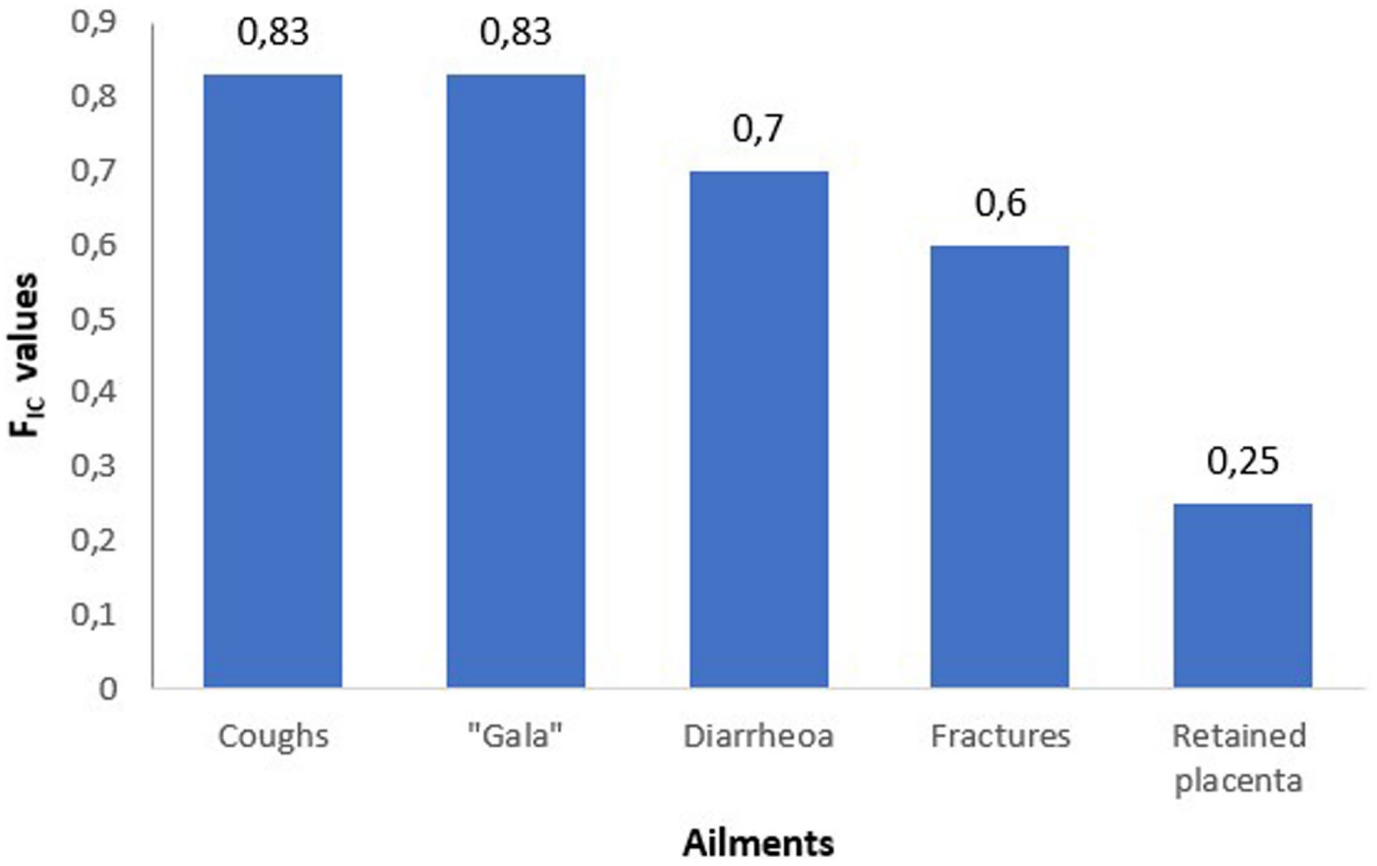
Informant consensus factor values for the treatment of some ailments in animals.

To determine medicinal plants that are culturally significant for treating animal diseases in the study area, the informant consensus factors were calculated (F_IC_) for six animal health diseases with a higher frequency of mention. The conditions were as follows: fractures; diarrhoea; *gala* (general malaise); retained placenta; coughs; and blood cleansing. The informant consensus factor values show agreement among participants for treatment with medicinal plants for five animal diseases. The highest F_IC_ values (between 0 and 1) were used to generate a column chart (Figure 4). It was observed that there is agreement among participants in the treatment of diarrhoea, *gala*, coughs, retained placenta, and fractures. The highest consensus values were for treating coughs using *Helichrysum caespititium* (phateyangaka) and *Senna italica* (sebetebete) for treating *gala* both at 0.83. *Senna italica* (sebetebete) is also a significant plant of choice for treating diarrhoea at FIC value 0.7. Treatment of fracture using *Vachellia karroo* (mookana) had a F_IC_ value 0.6. The lowest F_IC_ value was for treating retained placenta using *Boophone disticha* at 0.25 (1).

As stated above, *Senna italica* emerged as plant of cultural significance. This plant is recommended for treating calf diarrhoea, general malaise due to bile build-up (gala), gallsickness, retained placenta, abortions, gastrointestinal parasites, and blood cleansing. In the study by Van der Merwe et al. (22) among Batswana people of Madikwe, the plant was used to treat gallsickness, diarrhoea, general intestinal diseases, heartwater, anthrax, and pneumonia. Though the treatment of gallsickness and diarrhoea was the only similar indications in both studies, the multiple treatment indications of the plant in the two studies show the importance of the plant among the Batswana people. The findings of this study also concur with those of Gabalebatse et al. (6) who conducted a study in among Batswana of Botswana, in that study the plant was recommended for calf diarrhoea. The treatment of retained placenta found in the current study is a similar finding to the study by Moichwanetse et al. (27). Interestingly, the mention of *Senna italica* in other South African ethnoveterinary surveys is few, as shown in the review by McGaw et al. (7) despite the fact that the plant has many beneficial medicinal properties.

*Senna italica* has been scientifically tested to assess its medicinal potential and was discovered to possess different phytochemicals as reviewed by Masoko et al. (32). The plant is reported to possess alkaloids, quinines, anthraquinones and flavonoids. Furthermore, Dabai et al. (33) also determined the presence of steroids, alkaloids, and flavonoids. These compounds give *Senna italica* different medical effects that include anti-inflammatory, purgative, antiviral, antibacterial, antifungal, anti-tick and antioxidant medicinal properties (32, 34, 35). Batswana people likely discovered the plant’s broad-spectrum healing properties, which explains their trust in its medicinal value.

*Helichrysum caespititium* was another culturally important medicinal plant in the study area for the treatment of coughs caused by respiratory tract infections. This is not a surprise as the plant has similar uses in humans, the plant was reported by South Africans for use in treatment of respiratory tract infection symptoms such as cough, fever, blood in the sputum, flu (influenza) and chest complaints (36, 37). The plant extract is reported to exhibit antimicrobial activities during testing by different researchers (37) and found to possess inhibitory activity against drug-sensitive strain of *Mycobacterium tuberculosis* (36). Those finding validate the use of this plants by the indigenous communities in this study.

*Boophone disticha* was also found to be an important treatment of retained placenta in this study. A similar treatment indication for the plant as documented by Moichwanetse et al. (27). In addition, the plant was reported for use in cases of abortions in cattle by Van der Merwe et al. (22) and though not the same indication as found in the current study the findings shows that the plant is significant in treatment of reproductive problems among Batswana people. According to the review by Nair et al. (38), the plant is reported to contain a unique alkaloid constituents which are dividable into six structurally-diverse groups. These alkaloid constituents give the plant its antimicrobial, anti-inflammatory and anticancer activities. The participants in this study claimed that the plant has the ability to cause foetal membrane expulsion and cleanses the uterus. The cleansing ability can be validated by the antimicrobial activity of the plant (38). However, its ability to cause foetal membrane expulsions could not be substantiated at this point.

### 3.6 Symbolism in the Batswana names of medicinal plants

**Figure 5:**
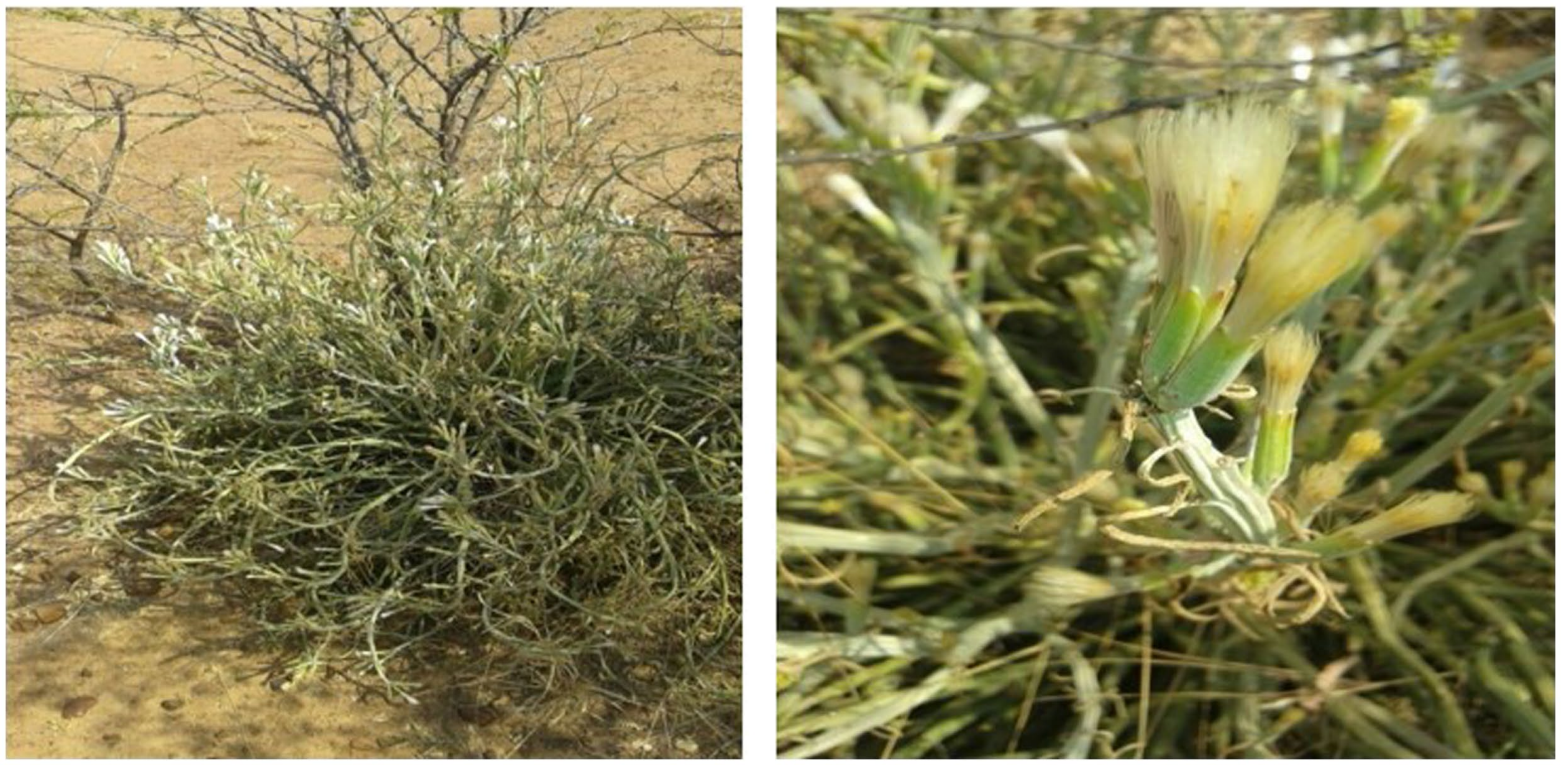
“Mosiama” *(Kleinia longiflora)* whose flowers have a feathery texture. Source: Researcher’s data.

Issues of semantics in the names of Batswana medicinal plants emerged during an interview with one of the participants. The researcher had mispronounced the word *phateyangaka* (*Helichrysum caespititium*) and the participant took time to explain the proper pronunciation and why the plant is referred to by this name. The participant indicated that the plant spreads out on the ground like a traditional reed mat (traditional carpet). Thus, it is referred to as a mat belonging to a traditional healer. The association with a traditional healer stem from the fact that the plant is very effective in healing. The best explanation of the symbolic naming of medicinal plants by Batswana people was as follows: “*Batswana people when naming medicinal plants, give them names that show the plant’s purpose in the body.*” A description of the names of some plants as provided by participants were as follows:

- “Mookana” (*Vachellia karroo*) is used to repair fractures either during *go tlhabela thobega* or as splints; its name, refers to a plant with the capacity to attract others to itself. This meant that when the plant is used to treat fractures, it would cause the broken fragments to pull towards each other, thus closing the gap of the fracture line faster.
- “Moologa” (*Croton gratissimus*) is used for fertility enhancement in livestock. The name of the plant refers to something that continuously overflows, indicating that when the plant is administered to animals, it would cause an unceasing overflow (production) of calves, kids and lambs.
- “Mosiama” (*Kleinia longiflora*) is a plant that has both physical and spiritual importance (Figure 5). The plant is used to repair fractures (*go tlhabela thobega*) and to cleanse animals and people after the death of a family member. *Mosiama* comes from a statement (*o a siamisa*) which means to correct an incorrect situation. The plant is essential in the cleansing rituals of family members and livestock after a funeral. The belief behind its use after funerals is that death spiritually disarranges the family (sefifi). The disarrangement can lead to bad luck and the death of other family members and livestock. To bring back spiritual balance, *mosiama* is used during cleansing rituals. The function of this plant during rituals is testimony of African people’s reliance in, trust of, and connectivity with their natural resources for both spiritual and physical healing.

This is the first report on the symbolic naming of ethnoveterinary medicinal plants in South Africa. The phenomenon is best explained by Possa and Khotso (39), who extensively explored the practice of naming of ethnobotanical plants among Basotho. Possa and Khotso (39) concluded that most vernacular medicinal plant names are a literal interpretation of the plant’s healing properties. Additionally, according to that study, a name can influence a situation or characteristics of a person or object. This sentiment is shared by the participants in the current study.

### 3.7 Trust in ethnoveterinary medicine

Participants were requested to indicate their confidence level for the medicinal plants they mentioned as ‘low’, ‘medium’, or ‘high’ confidence. Only one plant, *Babiana hypogaea* (*thuge*), used to treat goat diarrhoea was rated low by the informing participant. The participant indicated that the reason for the low confidence was because he was not so sure of its effectiveness and had never used it himself. Regarding the rest of the medicinal plants, participants had high confidence in their efficacy, and such confidence is best represented by the excerpt from a participant: *“I use traditional medicines for my livestock and that is why I was the first participant you were referred to in the village. I know these plants and I am always in the look out for them in the field when I am herding my animals. I use them and they are effective and swift to work.”*

Another participant expressed trust in EVM: *“I cannot remember all the remedies that I once knew but what I still remember, I still use, and they still work very well for me.”* In addition to the trust for EVM, some participants believed the remedies they used in the olden days were not too many because there were fewer diseases than currently. A participant expresses this sentiment as follows: *“Long ago, we did not have as many diseases as we do today. In fact, livestock products did not make a person sick like it happens these days with diseases such as gout. In the olden days, when a cow was sick, we would use traditional remedies and the following day, the animal would be as healthy as if it was never sick.”* The belief in ethnoveterinary medicine was echoed by all participants. One participant summed up this belief in the following excerpts: *“These remedies work and since you are a scientist, go and test them and you will come back and testify.”*

In South Africa, traditional healing is widely trusted and preferred by many indigenous communities. Studies indicate that around 70%–80% of black South Africans consult traditional healers for medical advice (7, 8), which highlights the people’s faith in this form of medicine. Similarly, a high level of trust and confidence is observed in the use of ethnoveterinary medicine (EVM) in South Africa (7, 15). For instance, a study conducted in the Eastern Cape Province by Masika et al. (40) showed that 89% of respondents trusted EVM. This trust in EVM is not only limited to South Africa but is also found in many farming communities throughout Africa, where some livestock keepers rely solely on ethnoveterinary medicine even when Western veterinary medicines are available (41).

### 3.8 Concerns for knowledge loss among the indigenous community

During the data collection process, the researcher noticed that the interviews with older participants always started slowly as they took their time to gather their thoughts and recollect their knowledge of ethnoveterinary medicine. Most of the older participants admitted to having forgotten some of the EVM knowledge that was practiced by their grandparents, parents, and themselves during their active farming days. Additionally, all participants agreed that the knowledge of ethnoveterinary medicine has been lost over time and continues to disappear. There were three factors identified as the cause of this phenomenon; namely, aging which leads to diminishing memories, availability of Western medicine, and disinterest of the younger generation. In the next sections, availability of Western medicine and disinterest of the younger generation.

#### 3.8.1 Availability of western medicines in local towns

During the interviews, a few participants mentioned that they had abandoned some EVM practices in favour of Western medicines and unfortunately, this is to the detriment of the retention of the knowledge. Participants cited Western medicines such as Terramycin (an antibiotic), Triatix (external parasite remedy), and Prodose (anti-helminthic) as medicines they used regularly. At the beginning of an interview with a participant, a statement was made that best describes why knowledge of EVM is being lost within the communities due to the availability of Western medicine: *“Honestly, I have forgotten most of the EVM remedies we used in the olden days. I remember the remedies I still use for my livestock, like sebetebete. But, I could show you all the Western medicine I currently use, including Terramycin and “dip.” My son buys them in bulk and brings them to me.”*

Several participants indicated that the interviews made them realise how much information they had already lost and were glad someone was taking an interest in the knowledge they possessed. One participant was particularly excited about their interview. When asked why, the following was declared, *“As I am speaking to you, it is like my brain is being opened and I remember a lot of things that we used for animal health care. I still use a lot of ethnoveterinary medicine but some practices I abandoned because of the availability of Western medicines.”* Another participant said a statement that shows the prejudice that is sometimes faced by all African traditional medicine including EVM: *“Medical indigenous knowledge is being lost because Western-trained practitioners like nurses discourage us from using them. They tell us to stop digging for medicinal plants and go to the clinics to seek medical attention.”*

#### 3.8.2 Lack of interest among the younger generation

A common concern among older participants was that their descendants’ disinterest in EVM would result in the loss of knowledge. That concern was best expressed by a participant who when asked if knowledge of EVM was shared with their children and grandchildren, the response was as follows; *“When we tell our children and grandchildren about traditional medicine for animals and any other olden practices, they laugh at us and say these practices are outdated, they also claim not to have time to learn such things.”* Another participant, further elaborated on the lack of interest in EVM as captured in the following statement: *“We send them (the descendants) to school and some of them even studied agriculture, understandably, they now prefer to use their Western knowledge for animal health care. They say traditional medicine for the treatment of animals is not scientific. So, what can we say to that?.”* However, some participants also acknowledged that if they were raised in modern times and never witnessed the efficacy of EVM, they would also probably prefer Western medicine as it is available at retail shops.

Participants stated that their learning of EVM as a necessary skill for farming and taught by parents or grandparents when they were young. This learning involved both theoretical and practical methods. A participant best expressed the sentiment in the following excerpt: *“Our parents would tell you about the remedies, show you the medicinal plants in the field and how to dig them out, and how to prepare the remedies. The next time the remedies are needed, they will send you to collect the plants and prepare the remedy yourself. That is how we learned these things.”* Even the youngest person in the study shared almost the same learning method. Indicating that the learning of ethnoveterinary medicine was through a series of theoretical and practical teachings, which led to the appreciation of the effectiveness of EVM. In the case of one participant, their childhood was described as a time full of learning ethnomedicine for both humans and animals from his father, a traditional healer. During that time, the philosophy of traditional healing and its application was learned through the father’s guidance.

It was also noted that older participants were more excited about the interviews than younger participants. The older generation indicated that the interviews allowed them to recall and share their knowledge. This sentiment is best captured by the excerpt from a participant as follows: “*You do well by asking these questions and recording the information because as for us, where we are going (death) is near and we will go with the knowledge.”*

The risk of losing knowledge of ethnoveterinary medicine over time is a concern that has been raised in several African ethnoveterinary publications, as reported by a review conducted by Wanzala et al. (41). The phenomenon is also reported by Luseba and Van der Merwe (21), Wanzala et al. (41), Yirga et al. (42), and Yineger et al. (43). Unfortunately, this study has confirmed the gravity of the situation and highlights the severity of the situation from the perspective of South African knowledge holders. Most of the study participants were elderly and concerned that their knowledge of ethnoveterinary medicine would be lost with their passing. Similar concerns were raised by studies by Gabalebatse et al. (6) in Botswana and Matekaire and Bwakura (44) in Zimbabwe. A study conducted by Wanzala et al. (45) among the Bukusu community in Kenya, revealed that the loss of knowledge in ethnoveterinary medicine is caused by various factors. Firstly, the death of individuals possessing ethnoveterinary knowledge without proper documentation. Secondly, the extinction of specific animal and plant species, as well as traditional practices. Lastly, the encroachment of modernisation and development on traditional and cultural life. These factors have led to a decline in the knowledge that is passed on to the next generation.

The lack of “formal” retention systems for ethnoveterinary medicine is one of the major contributors to the loss of knowledge in this field. Unlike its counterpart the human ethnomedicine, which is usually retained and protected by traditional healers (8), ethnoveterinary medicine does not have that privilege. In ethnomedicine, knowledge is passed down from generation to generation by training of new traditional healer initiates, known as “matwasane” (8). It is worth noting that most traditional healers did not acquire knowledge of ethnoveterinary medicine through their training. Rather, they learn it from their parents or grandparents (12). While some of the treatment methods used for animals are adaptations of those used in humans, the traditional healer who participated in the study confirmed that most of their knowledge of ethnoveterinary medicine came from parents.

Integration of Western veterinary medicine and ethnoveterinary medicine is crucial for retaining the knowledge of ethnoveterinary medicine (7). Integrative healthcare should also involve the inclusion of traditional medicine in education. Countries such as China, India, the Republic of Korea, and Vietnam have successfully integrated traditional medicine into higher education and offer relevant qualifications at university level (46). Despite attempts made by the South African government to integrate the two health systems since the 1990s, integration has not yet been optimally achieved (47). Achieving an integrative health system would require collaborative efforts of different stakeholders and huge financial commitments (8). The integration of ethnoveterinary medicine into the curriculum of higher learning offers a promising prospect to resolve the issue of loss of this indigenous knowledge and the promotion of a sustainable agriculture. As a result, the Faculty of Veterinary Science at the University of Pretoria (7) and the Department of Animal Health at North West University have integrated an ethnoveterinary medicine module into their curriculums.

### 3.9 Conservation challenges of medicinal plant species

In this study, the issue of conservation of medicinal plant species, the seriousness of the situation was highlighted by two participants during a field walk. One participant expressed concern that other plants were difficult to find due to traditional healers depleting the fields of these plants. They blamed the improper collection methods of traditional healers from outside the village who harvest without considering plant conservation. The second incidence was with the plant *Withania somnifera* (modikasope). After the interview, the participant was reluctant to identify the plant in the field. Instead, the participant collected a fresh part of the root and some of its arial parts and delivered them to the researchers’ office. On enquiry, the researcher was informed that the plant had become very scarce as traditional healers were depleting it. Thus, the participant did not want to reveal the location of the plant in the village to prevent its extinction. The challenge of medicinal plant species conservation was not unique to *Withania somnifera*. During other field walks with participants, plants such as *Boophone disticha, Cadaba aphylla and Drimia sanguinea* could not be identified in the fields where they were expected to be found. In such cases, the plant had to be “hunted” in other locations. Fortunately, all plants that were documented in this study were found.

Plant conservation is a critical issue in the use of medicinal plants worldwide, including South Africa (48). The increasing use of these plants has led to overexploitation, which poses a threat to biodiversity (49). To address this issue, Botha et al. (48) suggest that cultivating important plant species in various areas of South Africa could help preserve them. They also recommend implementing local-level management interventions in the harvesting and marketing of medicinal plants, as well as regional cooperation in management strategies and policies for harvesting plants for national and international markets. These recommendations, particularly cultivation and local-level management interventions, could be considered to conserve plants within Mahikeng Local Municipality.

## 4 Conclusion

This study documented the use of ethnoveterinary medicinal plants by Batswana people in Barolong boo Tshidi land, located in the Mahikeng Local Municipality. The study revealed several important themes, including the broad-spectrum curative properties of some plants, the trust in ethnoveterinary medicine, the symbolism in Setswana plant names, and the concern over the loss of knowledge of ethnoveterinary medicine due to Westernisation and acculturation of the younger generation. This study recommends urgent surveys to document EVM in unexplored regions of North West Province. Furthermore, efforts to integrate ethnoveterinary medicine into applicable basic and tertiary education should continue.

## Data availability statement

The original contributions presented in the study are included in the article/supplementary material, further inquiries can be directed to the corresponding author.

## Ethics statement

The studies involving humans were approved by North-West University Research Ethics Committee. The studies were conducted in accordance with the local legislation and institutional requirements. The participants provided their written informed consent to participate in this study.

## Author contributions

RN: Conceptualization, Formal analysis, Funding acquisition, Investigation, Methodology, Project administration, Writing – original draft, Writing – review & editing. SM: Conceptualization, Funding acquisition, Supervision, Writing – review & editing. MM: Supervision, Writing – review & editing. WO-M: Supervision, Writing – review & editing. MI: Investigation, Writing – review & editing.
